# Impact of Social Isolation, Physician-Patient Communication, and Self-perception on the Mental Health of Patients With Cancer and Cancer Survivors: National Survey Analysis

**DOI:** 10.2196/45382

**Published:** 2023-04-07

**Authors:** Avishek Choudhury

**Affiliations:** 1 Industrial and Management Systems Engineering Benjamin M Statler College of Engineering and Mineral Resources West Virginia University Morgantown, WV United States

**Keywords:** cancer communication, cancer stigma, mental health, social isolation, cancer survivorship, patient-centeredness

## Abstract

**Background:**

Cancer is perceived as a life-threatening, fear-inducing, and stigmatized disease. Most patients with cancer and cancer survivors commonly experience social isolation, negative self-perception, and psychological distress. The heavy toll that cancer takes on patients continues even after treatment. It is common for many patients with cancer to feel uncertain about their future. Some undergo anxiety, loneliness, and fear of getting cancer again.

**Objective:**

This study examined the impact of social isolation, self-perception, and physician-patient communication on the mental health of patients with cancer and cancer survivors. The study also explored the impact of social isolation and physician-patient communication on self-perception.

**Methods:**

This retrospective study used restricted data from the 2021 Health Information National Trends Survey (HINTS), which collected data from January 11, 2021, to August 20, 2021. We used the partial least squares structural equation modeling (PLS-SEM) method for data analysis. We checked for quadratic effects among all the paths connecting social isolation, poor physician-patient communication, mental health (measured using the 4-item Patient Health Questionnaire [PHQ-4]), and negative self-perception. The model was controlled for confounding factors such as respondents’ annual income, education level, and age. Bias-corrected and accelerated (BCA) bootstrap methods were used to estimate nonparametric CIs. Statistical significance was tested at 95% CI (2-tailed). We also conducted a multigroup analysis in which we created 2 groups. Group A consisted of newly diagnosed patients with cancer who were undergoing cancer treatment during the survey or had received cancer treatment within the last 12 months (receipt of cancer treatment during the COVID-19 pandemic). Group B consisted of respondents who had received cancer treatment between 5 and 10 years previously (receipt of cancer treatment before the COVID-19 pandemic).

**Results:**

The analysis indicated that social isolation had a quadratic effect on mental health, with higher levels of social isolation associated with worse mental health outcomes up to a certain point. Self-perception positively affected mental health, with higher self-perception associated with better mental health outcomes. In addition, physician-patient communication significantly indirectly affected mental health via self-perception.

**Conclusions:**

The findings of this study provide important insights into the factors that affect the mental health of patients with cancer. Our results suggest that social isolation, negative self-perception, and communication with care providers are significantly related to mental health in patients with cancer.

## Introduction

### Background

Cancer is perceived as a life-threatening, fear-inducing, and stigmatized disease [1-3]. Cancer diagnosis and treatment require a longitudinal and systematic approach involving a multidisciplinary care team, including pathologists, radiologists, oncologists, nurses, and social workers. The members of a care team often perform tasks at broadly two levels: (1) clinical activities and (2) nonclinical activities. Most of the efforts and resources of the care team are invested in augmenting the clinical activities that directly improve cancer detection. The nonclinical tasks involve verbal and nonverbal communication with the patients and other team members, which needs further development.

Textbox 1 presents a simplified version of the overall cancer care process (from diagnosis to treatment to aftercare) from a patient’s perspective. It should be noted that the simplified version provides a broad understanding of the journey of a typical patient with cancer. The process might differ across different health care establishments. Once clinically diagnosed with cancer, a patient is likely to undergo complex emotional experiences and face challenges in handling the bad news, selecting treatment options, dealing with the social isolation and stigma, and, most importantly, performing all the patient tasks (eg, comprehending diagnosis, traveling, following treatment protocols, communicating with the care team, handling self-care activities, managing finances, seeking help from family, and making arrangements to support dependents) throughout the treatment process. Some even believe that the cancer treatment is worse than the ailment [2]. The heavy toll that cancer takes on patients continues even after treatment. It is common for many patients with cancer to feel uncertain about their future. Some undergo anxiety, loneliness, and fear of getting cancer again. They even experience fatigue, difficulty sleeping, persistent pain from neuropathy, and emotional distress. The struggles worsen with age [4] and low health literacy [5].

**A simplified version of the cancer care pathway in sequential order from a patient’s perspective.**

- Cancer suspicion and diagnosis: at this stage, the patient feels discomfort and visits their care provider for a medical checkup. The care provider prescribes cancer diagnostic tests.
- Receiving the cancer *bad news*: this is the moment when the patient, after waiting days or weeks to get the diagnosis results, visits the clinic to receive the cancer *bad news*.
- Comprehending the diagnosis, care options, and next steps: on the same day, after receiving the bad news, the patient must understand their diagnosis, cancer severity, treatment options, and next steps. It should be noted that the patient is undergoing negative emotions from the diagnosis.
- Communicating with the oncologist: on the same day or shortly afterward, the patient, along with the family (if any), must speak with the oncologist to discuss the treatment in detail.
- Communicating with the care team, including social worker: depending on the patient’s needs, they must speak with other care team members about the support they might need during and after treatment.
- Scheduling appointments for the treatment: at this stage, based on availability, treatment appointments are scheduled.
- Receiving the treatment: this is the period (several months) during which the patient must travel, receive the cancer treatment, and adhere to any clinical recommendation. This is when patients gradually become isolated from society and their usual day-to-day activities.
- Recovering from the treatment: this is when the patient recovers physically and mentally from the often painful treatment process.
- Trying to get back to normal life: this is when the patient voluntarily engages with other cancer survivors or patients with cancer on dedicated digital venues to share their journey. Although still scared of getting cancer again, they try to gradually return to their normal life.

### Study Hypotheses

Most patients with cancer and cancer survivors commonly experience social isolation, negative self-perception, and psychological distress [6-9]. Prior studies have explored how cancer induces these challenges [10-13]; for example, studies have been conducted to capture the negative impact of pain and exhaustion associated with cancer treatments (chemotherapy and radiation) on a patient’s mental health [14-16]. However, there is a lack of evidence capturing the impact of social isolation and self-perception on psychological distress in patients with cancer. Therefore, we explore the association between social isolation and mental health, hypothesizing that increased social isolation will hinder the mental health of patients with cancer (hypothesis 1).

The potential impact of social isolation among patients with cancer and cancer survivors can extend beyond mental health concerns to the point where it can distort their self-perception [17-19]. When patients with cancer experience social isolation for an extended time for any given reason, be it a disrupted lifestyle or limited physical capability, they start developing negative perceptions about themselves, particularly negative perceptions of their general health and self-care ability. Besides, cancer treatment can often lead to physical changes, such as hair loss, weight changes, or scarring. These changes can be difficult for patients to adjust to and can exacerbate negative self-perceptions. The extent to which social isolation contributes to this negative self-perception is not yet studied. To address this gap, we explore the association between social isolation and negative self-perception of patients with cancer and cancer survivors, hypothesizing that increasing social isolation will encourage (increase) negative self-perception (hypothesis 2).

**Figure 1:**
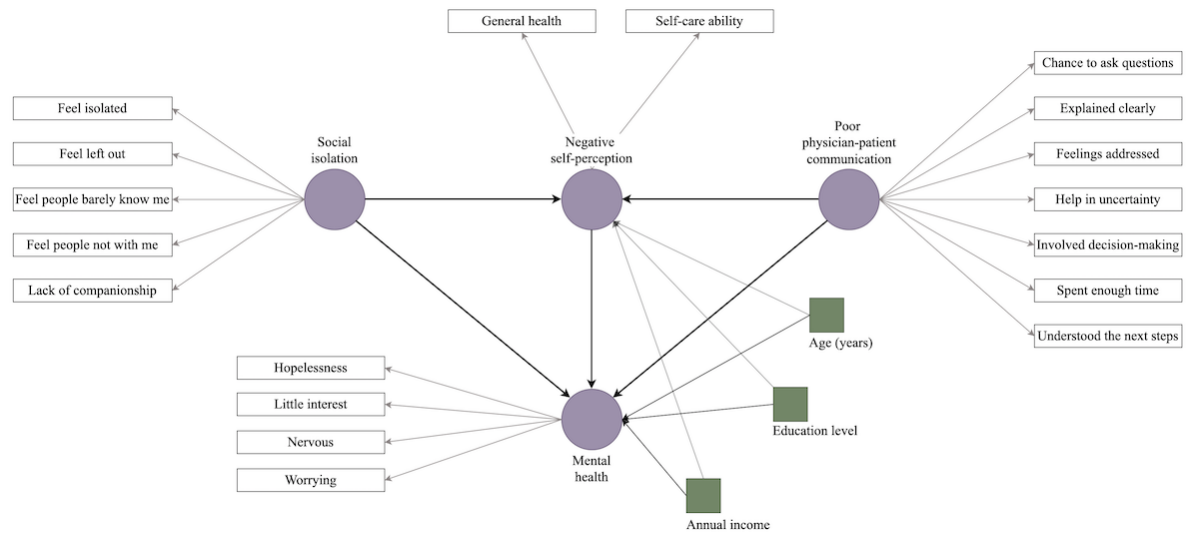
Conceptual framework illustrating the impact of social isolation and poor physician-patient communication on mental health (measured using the 4-item Patient Health Questionnaire for Anxiety and Depression [PHQ-4]) and negative self-perception of patients with cancer.

Effective physician-patient communication is essential in cancer care for several reasons. It ensures patient satisfaction, facilitates shared decision-making, and helps patients with cancer to understand their diagnosis, treatment options, and prognosis. Overall, effective communication can relieve patients from some of the mental and emotional burdens of the care process and help to make the process more patient centered. By contrast, poor communication may lead to confusion and misunderstandings, contributing to patient anxiety and uncertainty. Patients may feel that their concerns are not heard or addressed, leading to frustration and mistrust. However, there is a lack of evidence confirming the potential impact of communication on the mental health of patients with cancer. Therefore, this study explores the association between patients’ poor communication with the care provider and their mental health, hypothesizing that poor physician-patient communication negatively affects their mental health (hypothesis 3). We also explore the impact of poor communication on patients’ negative self-perception and hypothesize that poor physician-patient communication will increase the negative self-perception of patients (hypothesis 4). Figure 1 illustrates the interactions explored in this study. Our study will contribute to the existing body of knowledge by providing a more in-depth understanding of the role of social isolation, self-perception, and physician-patient communication in the mental health of patients with cancer.

## Methods

### Ethics Approval and Data Source

The study was approved by the institutional review board of West Virginia University, Morgantown, West Virginia, United States (2212691613). The 2021 Health Information National Trends Survey (HINTS) deidentified data were obtained and analyzed after approval from the National Cancer Institute (NCI). HINTS is a nationally representative survey of adults in the United States that aims to assess attitudes, behaviors, and knowledge related to cancer and cancer prevention [20].

### Survey Instruments and Latent Constructs

In a 2021 pilot program, the NCI administered this survey to oversample cancer survivors using 3 cancer registries from the Surveillance, Epidemiology, and End Results (SEER) program. The pilot program, called HINTS-SEER, was designed to provide a larger sample of cancer survivors for HINTS analyses. HINTS-SEER data were collected from January 11, 2021, to August 20, 2021 [21]. According to HINTS data, 1234 respondents completed the survey. The HINTS service considers a questionnaire to be complete if at least 80% of the questions in each section of the survey are answered [21]. For our research, we handled missing data using the pairwise deletion method.

We used 18 observed variables from the HINTS-SEER survey to feed the proposed conceptual model. Questions from these 18 items were grouped to form 4 latent reflective constructs (Table 1). All 4 latent constructs’ convergent, reliability, and discriminant validity were validated. The respondents’ *mental health* was measured using the 4-item Patient Health Questionnaire (PHQ-4), which is a brief self-report measure of depression and anxiety used to assess the presence and severity of these conditions [22]. The PHQ-4 consists of 2 questions, 2 of which are designed to assess symptoms of depression (2-item Patient Health Questionnaire [PHQ-2]) and 2 of which are designed to assess symptoms of anxiety (2-item Generalized Anxiety Disorder scale [GAD-2]). Each question asks the respondent to rate the frequency of specific symptoms over the past 2 weeks using a 4-point Likert scale ranging from *not at all* to *nearly every day* [22,23].

The construct of *social isolation* was measured using questions from the Patient-Reported Outcomes Measurement Information System (PROMIS) social isolation instrument [24]. This is a self-report measure used to assess the extent to which an individual experiences feelings of loneliness and social disconnection [24]. The PROMIS social isolation instrument consists of 4 items designed to assess both the quantity and quality of an individual’s social interactions and the perceived support they receive from their social network. Each item on the PROMIS social isolation instrument asks the respondent to rate the frequency of specific feelings and experiences related to social isolation using a 5-point Likert scale ranging from *never* to *always* [24]. We added another question (*How often do you feel you lack companionship?*) to improve its convergence.

Seven other questions were combined to determine the quality of physician-patient communication (PPC). The PPC scale is a tool used to assess the quality of communication between patients and health care providers [25]. The scale consists of 7 questions that ask the respondent to rate the extent to which they agree or disagree with statements about their communication with their health care provider. Each question is rated on a 4-point Likert scale ranging from *always* to *never*.

Similarly, questions regarding patients’ self-health perception and ability to manage self-care were combined to measure their self-perception. In the context of this study, self-perception is a multidimensional construct that refers to an individual’s beliefs, attitudes, and evaluations of themselves and their abilities. In the context of health, self-perception may include an individual’s beliefs and attitudes about their health, their ability to manage their health and well-being, and their perceived control over their health outcomes. General health perception, which refers to an individual’s overall perceptions of their health, is an essential aspect of self-perception in health. Research has shown that an individual’s general health perception is related to a range of health behaviors, including adherence to treatment regimens, engagement in health-promoting behaviors, and use of health care services. Perception of self-care, which refers to an individual’s beliefs and attitudes about their ability to take care of themselves, is also an important aspect of self-perception in the context of health. Individuals who perceive themselves as able to manage their health and well-being effectively may be more likely to engage in health-promoting behaviors and seek appropriate health care services when needed. Combining measures of general health perception and perception of self-care can provide a more comprehensive understanding of an individual’s self-perception in the context of their health.

**Table 1:** Reliability and validity of the latent constructs.

Construct	Cronbach *α* (>.70^a^)	Composite reliability (>0.70^a^)		AVE^b^ (>0.50^c^)
		*ρ*_a_	*ρ*_c_	
Mental health (PHQ-4^d^)	.88	0.89	0.88	0.65
Social isolation	.89	0.90	0.89	0.63
Poor physician-patient communication	.92	0.92	0.92	0.61
Negative self-perception	.72	0.72	0.72	0.57

^a^Adequate fit.

^b^AVE: average variance extracted.

^c^Acceptable fit.

^d^PHQ-4: 4-item Patient Health Questionnaire.

### Structural Equation Modeling

We used partial least squares structural equation modeling (PLS-SEM) to explore the proposed conceptual framework [26]. This method allows the simultaneous estimation of multiple and interrelated dependent relationships between variables and latent constructs [26]. We used the bootstrapping method with 5000 subsamples and controlled for possible confounding factors such as respondents’ annual income, education level, and age. Bias-corrected and accelerated (BCA) bootstrap methods were used to estimate nonparametric CIs. Statistical significance was tested at 95% CI (2-tailed).

During the recent COVID-19 pandemic, the emotional distress and the number of deaths were significant factors responsible for increased mental health problems and social isolation across the globe. Many were scared and uncertain about the impact of SARS-CoV-2 on them or their families. This emotional distress was even more ingrained among patients with cancer. Many cancer treatments and consultations were delayed because of the unmanageable workload in the health care industry. The government-imposed lockdowns worldwide also contributed to the social isolation of many individuals, and patients with cancer were not an exception. Given these circumstances, that is, increased mental health problems, social isolation, and overwhelmed health care industry, it is acceptable to assume that the association among social isolation, mental health, physician-patient communication, and self-perception in patients with cancer would be significantly different during the pandemic than during other times. Therefore, we conducted a multigroup analysis (MGA) to test this assumption.

In structural equation modeling (SEM), MGA is a statistical technique used to compare a structural model’s fit across different groups or subpopulations [27]. The MGA allows researchers to test whether the same model fits equally well across other groups or whether there are significant differences in the relationships among variables between 2 groups [27]. In this MGA, we created 2 groups. Group A consisted of newly diagnosed patients with cancer who were undergoing cancer treatment during the survey or had received cancer treatment within the last 12 months, that is, receipt of cancer treatment during the COVID-19 pandemic. Group B consisted of respondents who had received cancer treatment between 5 and 10 years previously, that is, receipt of cancer treatment before the COVID-19 pandemic.

Finally, as an additional analysis, we tested for possible curvilinear effects. We checked for quadratic effects (QEs) [28] among all the paths connecting *social isolation*, *poor physician-patient communication*, *mental health* (measured using PHQ-4), and *negative self-perception*.

## Results

### Overview

Table 2 presents the statistics regarding the sociodemographic variables of the participants. Questions from these 18 items were grouped to form four latent reflective constructs as shown in Table 1: (1) social isolation, (2) negative self-perception, (3) poor physician-patient communication, and (4) mental health. Confirmatory factor analysis was performed to analyze their psychometric properties. All factor loadings were noted to be >0.50. The model fit was evaluated on the standardized root mean square residual (SRMR), an absolute measure of fit that is indicative of the standardized difference between the observed correlation and the predicted correlation. SRMR <0.080 is considered a good fit (observed=0.046). The constructs’ reliability and validity were determined using Cronbach *α*, composite reliability (*ρ*_a_ and *ρ*_c_), and the average variance extracted (AVE). The discriminant validity was measured using the heterotrait-monotrait (HTMT) ratio. All HTMT ratios were <0.85, indicating reliable discriminant validity. In addition, we checked for multicollinearity using the variance inflation factor (VIF) and did not find any evidence of multicollinearity. All VIF values were substantially <2.5, ranging between 1.03 and 1.7.

**Table 2:** Participant characteristics and demographics.

		Received cancer treatment, n (%)				
		Newly diagnosed	Cancer survivors			
		During survey, n (%)	<1 year previously, n (%)	Between 1 and 5 years previously, n (%)	Between 5 and 10 years previously, n (%)	>10 years previously, n (%)
**Sex**						
	Male (n=495)	62 (12.5)	30 (6.1)	119 (24)	109 (22)	174 (35.2)
	Female (n=580)	67 (11.6)	19 (3.3)	115 (19.8)	131 (22.6)	248 (42.8)
**Age group (years)**						
	18 to 34 (n=8)	0 (0)	0 (0)	2 (25)	3 (37.5)	3 (37.5)
	35 to 49 (n=31)	3 (9.7)	1 (3.2)	10 (32.3)	9 (29)	8 (25.8)
	50 to 64 (n=224)	26 (11.6)	5 (2.2)	55 (24.6)	60 (26.8)	78 (34.8)
	65 to 75 (n=373)	40 (10.7)	22 (5.9)	91 (24.4)	78 (20.9)	142 (38.1)
	≥75 (n=432)	58 (13.4)	21 (4.9)	75 (17.4)	88 (20.4)	190 (44)
**Education level**						
	Less than high school (n=30)	3 (10)	2 (6.7)	6 (20)	8 (26.7)	11 (36.7)
	High school graduate (n=130)	22 (16.9)	7 (5.4)	25 (19.2)	34 (26.2)	42 (32.3)
	College (n=288)	37 (12.8)	13 (4.5)	66 (22.9)	72 (25)	100 (34.7)
	Bachelor’s degree (n=297)	35 (11.8)	10 (3.4)	67 (22.6)	53 (17.8)	132 (44.4)
	Postbaccalaureate degree (n=327)	32 (9.8)	17 (5.2)	70 (21.4)	73 (22.3)	135 (41.3)
**Employment status**						
	Employed full time (n=204)	14 (6.9)	6 (2.9)	53 (26)	66 (32.4)	65 (31.9)
	Employed part time (n=60)	5 (8.3)	2 (3.3)	15 (25)	11 (18.3)	27 (45)
	Homemaker (n=40)	6 (15)	3 (7.5)	4 (10)	12 (30)	15 (37.5)
	Student (n=2)	0 (0)	0 (0)	1 (50)	1 (50)	0 (0)
	Retired (n=690)	89 (13)	32 (4.6)	139 (20.1)	131 (19)	299 (43.3)
	Disabled (n=38)	10 (26.3)	4 (10.5)	9 (23.7)	10 (26.3)	5 (13.2)
	Unemployed <1 year (n=10)	1 (10)	0 (0)	4 (40)	2 (20)	3 (30)
	Unemployed >1 year (n=10)	1 (10)	0 (0)	3 (30)	3 (30)	3 (30)
	Other (n=10)	2 (20)	1 (10)	4 (40)	1 (10)	2 (20)
**Race**						
	Non-Hispanic White (n=815)	99 (12.1)	37 (4.5)	180 (22.1)	185 (22.7)	314 (38.5)
	Non-Hispanic Black (n=14)	2 (14.3)	0 (0)	2 (14.3)	2 (14.3)	8 (57.1)
	Hispanic (n=115)	13 (11.3)	4 (3.5)	24 (20.9)	24 (20.9)	50 (43.5)
	Non-Hispanic Asian (n=61)	5 (8.2)	3 (4.9)	15 (24.6)	13 (21.3)	25 (41)
	Non-Hispanic other, n=10	3 (30)	1 (10)	1 (10)	3 (30)	2 (20)
**Annual household income (US $)**						
	<9999 (n=21)	3 (14.3)	3 (14.3)	2 (9.5)	1 (4.8)	12 (57.1)
	10,000 to 14,999 (n=38)	5 (13.2)	3 (7.9)	7 (18.4)	9 (23.7)	14 (36.8)
	15,000 to 19,999 (n=28)	5 (17.9)	1 (3.6)	6 (21.4)	9 (32.1)	7 (25)
	20,000 to 34,999 (n=123)	25 (20.3)	3 (2.4)	18 (14.6)	31 (25.2)	46 (37.4)
	35,000 to 49,999 (n=120)	9 (7.5)	8 (6.7)	27 (22.5)	26 (21.7)	50 (41.7)
	50,000 to 74,999 (n=177)	25 (14.1)	10 (5.6)	55 (31.1)	34 (19.2)	53 (29.9)
	75,000 to 99,000 (n=178)	19 (10.7)	5 (2.8)	35 (19.7)	40 (22.5)	79 (44.4)
	100,000 to 199,000 (n=272)	32 (11.8)	12 (4.4)	57 (21)	57 (21)	114 (41.9)
	≥200,000 (n=130)	9 (6.9)	5 (3.8)	29 (22.3)	35 (26.9)	52 (40)

### Failure to Reject Hypotheses

Table 3 shows significant direct, indirect, and total effects of social isolation on mental health, failing to reject hypothesis 1. In other words, increasing social isolation will negatively affect mental health. The finding of a significant indirect effect of social isolation on mental health via negative self-perception, as indicated by a negative coefficient (−0.08) and *P*<.001, suggests that social isolation may influence mental health in patients with cancer through its effect on negative self-perception. The negative coefficient for the indirect effect indicates that higher levels of social isolation are associated with reduced mental health outcomes through their impact on negative self-perception. This finding suggests that social isolation may have a risk effect or harmful effect on mental health in patients with cancer, potentially by increasing negative self-perception.

In addition, the QE was also significant between these 2 constructs. The significant QE indicates a statistically significant curvilinear relationship between social isolation and mental health in patients with cancer. The quadratic term (−0.136x^2^) represents the curvilinear relationship between the 2 variables. The negative coefficient indicates that the relationship is stronger at very high or very low levels of social isolation and weaker at moderate levels. The linear term (−0.321) represents the overall trend in the relationship between the 2 variables, with a negative coefficient indicating that mental health decreases as social isolation increases. It is important to remember that this equation represents the overall trend in the relationship between social isolation and mental health, but individual patients may not necessarily follow this trend. In other words, the QE suggests a threshold or optimal level of social isolation associated with better mental health outcomes. This optimal level may differ for individuals, depending on their personalities, coping strategies, and support networks; for instance, some patients with cancer may find that a certain degree of social isolation allows them to focus on their needs, engage in self-reflection, and develop a sense of independence and self-reliance, which can help to reduce mental distress. By contrast, too much social isolation may lead to feelings of loneliness, helplessness, and despair, which can negatively affect mental health.

The QE implies that interventions to reduce social isolation in patients with cancer should consider the complex and curvilinear relationship between social isolation and mental health. This may involve tailoring interventions to individual needs, providing different levels and types of social support, and encouraging patients to develop a sense of control and agency over their social relationships.

We observed significant direct and total effects of social isolation and negative self-perception. A significant direct effect of social isolation on negative self-perception with a coefficient of 0.36 would indicate that an increase in social isolation is associated with an increase in negative self-perception and vice versa. Therefore, we fail to reject hypothesis 2.

We did not observe any significant direct effect of poor communication on patient mental health; however, specific indirect and total effects were significant, failing to reject hypothesis 3. The finding of a significant indirect effect of poor physician-patient communication on the mental health of patients with cancer via negative self-perception, as indicated by a negative coefficient (−0.03) and a statistically significant *P* value, suggests that poor physician-patient communication may influence the mental health of patients with cancer through its effect on negative self-perception. The negative coefficient for the indirect effect implies that higher levels of communication are associated with better mental health outcomes through their effect on negative self-perception. This finding suggests that poor physician-patient communication may protect mental health in patients with cancer, potentially by reducing negative self-perception. poor physician-patient communication also had a significant direct and total effect on negative self-perception, implying that patients who experience inadequate communication with their care team will develop negative self-perception. Therefore, we fail to reject hypothesis 4.

**Figure 2:**
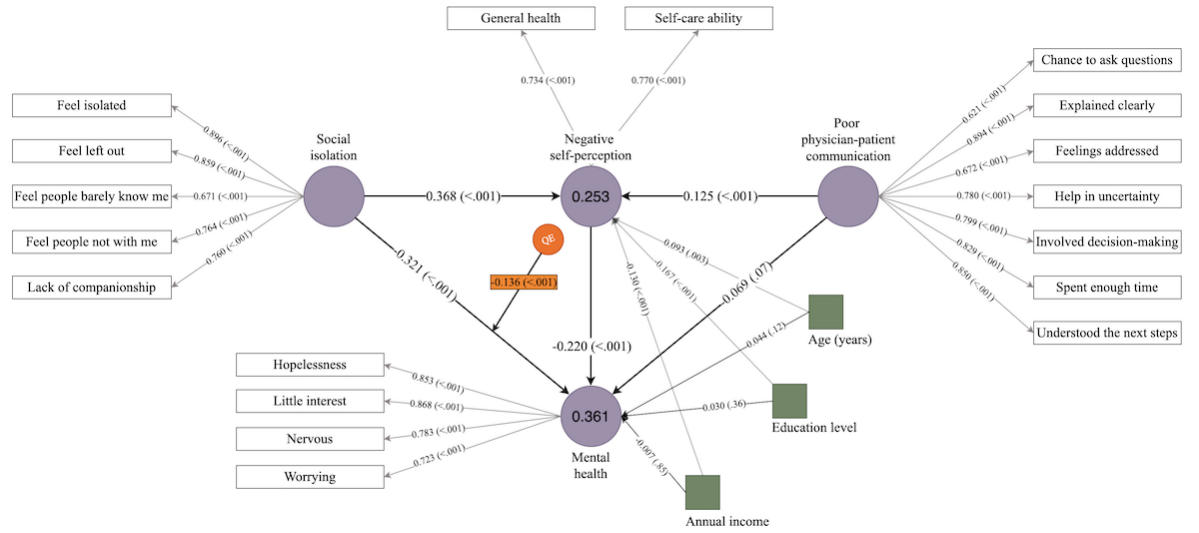
Structural framework illustrating direct and indirect relationships among social isolation, mental health (measured using the 4-item Patient Health Questionnaire for Anxiety and Depression [PHQ-4]), poor physician-patient communication, and negative self-perception. The model shows the path coefficient and its significance. The observed factors indicated by square boxes are the control variables, and the values on the 2 endogenous latent constructs are the R2 values. QE: quadratic effect.

In addition, the model identified a significant direct effect of negative self-perception on mental health, where increasing negative self-perception would hinder a patient’s mental health. The control variables, including age, annual income, and education, had no significant effect on mental health, but the indirect and total effects were significant. The direct effects of the control variables on negative self-perception were also significant. Patients with higher education and income had better self-perception (better perception of general health and better ability for self-care). Contrastingly, negative self-perception was found to increase with age. Figure 2 illustrates the final model.

**Table 3:** Standardized direct, indirect, and total effects.

Paths		Estimate (β)	Standardized mean estimate	2-tailed *t* test	*P* value
**Direct effects**					
	Social isolation → negative self-perception	.36	0.03	9.86	<.001
	Social isolation → mental health	−.32	0.04	6.67	<.001
	QE^a^ (social isolation) → mental health	−.13	0.03	3.64	<.001
	Poor communication with care provider → negative self-perception	.12	0.03	3.25	.001
	Poor communication with care provider → mental health	−.07	0.03	1.82	.07
	Negative self-perception → mental health	−.22	0.04	5.16	<.001
	Age → mental health	.04	0.02	1.53	.12
	Age → negative self-perception	.09	0.03	3.03	.003
	Annual income → mental health	−.01	0.04	0.18	.86
	Annual income → negative self-perception	−.13	0.03	3.54	<.001
	Education level → mental health	.03	0.03	0.91	.36
	Education level → negative self-perception	−.16	0.03	4.85	<.001
**Specific indirect effects**					
	Poor communication with care provider → negative self-perception → mental health	−.03	0.01	2.60	.009
	Social isolation → negative self-perception → mental health	−.08	0.02	4.72	<.001
	Age → negative self-perception → mental health	−.02	0.01	2.55	.01
	Education level → negative self-perception → mental health	.03	0.01	3.41	.001
	Annual income → negative self-perception → mental health	.02	0.01	2.86	.004
**Total effects**					
	Social isolation → mental health	−.40	0.47	8.79	<.001
	Social isolation → negative self-perception	.36	0.03	9.86	<.001
	QE (social isolation) → mental health	−.13	0.03	3.64	<.001
	Poor communication with care provider → mental health	−.10	0.04	2.57	.01
	Poor communication with care provider → negative self-perception	.13	0.04	3.26	.001
	Negative self-perception → mental health	−.22	0.04	5.16	<.001
	Age → mental health	.02	0.02	0.80	.42
	Age → negative self-perception	.09	0.03	3.02	.003
	Annual income → mental health	.02	0.03	0.60	.54
	Annual income → negative self-perception	−.13	0.03	3.54	<.001
	Education level → mental health	.06	0.03	2.05	.04
	Education level → negative self-perception	−.16	0.03	4.85	<.001

^a^QE: quadratic effect.

### MGA Results

Table 4 shows the conceptual framework across two groups: (1) group A consisted of newly diagnosed patients with cancer who were undergoing cancer treatment during the survey or had received cancer treatment within the last 12 months, that is, receipt of cancer treatment during the COVID-19 pandemic; and (2) group B consisted of respondents who had received cancer treatment between 5 and 10 years previously, that is, receipt of cancer treatment before the COVID-19 pandemic. We noted that the direct effect of negative self-perception on mental health was significantly higher among patients treated before the COVID-19 pandemic (group B). Similarly, the indirect effect of social isolation on mental health was significantly higher among patients who had received cancer treatment before the COVID-19 pandemic. We did not observe any significant differences in other effects.

**Table 4:** A multigroup analysis of patients with cancer receiving treatment during and before the COVID-19 pandemic.

Paths		Estimate (β; group A-B)		*t* test (group A-B)	*P* value (group A-B)
**Direct effects**					
	Social isolation → negative self-perception	.01		0.09	.92
	Social isolation → mental health	.11		0.92	.36
	Poor communication with care provider → negative self-perception	−.13		1.24	.24
	Poor communication with care provider → mental health	.05		0.56	.58
	Negative self-perception → mental health	−.21		2.23	.03
	Age → mental health	.03		0.35	.72
	Age → negative self-perception	−.07		0.79	.43
	Annual income → mental health	.03		0.34	.73
	Annual income → negative self-perception	.03		0.33	.74
	Education level → mental health	−.02		0.22	.83
	Education level → negative self-perception	.09		1.02	.31
**Specific indirect effects**					
	Poor communication with care provider → negative self-perception → mental health	−.01	0.18		.86
	Social isolation → negative self-perception → mental health	−.06	2.01		.04
	Age → negative self-perception → mental health	−.01	0.69		.49
	Education level → negative self-perception → mental health	.01	0.67		.50
	Annual income → negative self-perception → mental health	.02	1.22		.22

## Discussion

### Principal Findings

The findings of this study provide important insights into the factors that affect the mental health of patients with cancer. Our results suggest that social isolation, negative self-perception, and communication with care providers are significantly related to mental health in patients with cancer.

It is common for patients with cancer to experience mental health challenges such as anxiety, depression, and distress, which can be further exacerbated by social isolation. Several studies have assessed the impact of social isolation on mental health; for instance, a 2021 study reported a significant association between social isolation and loneliness in patients with cancer during the COVID-19 pandemic [29]. The study also acknowledged the correlation between loneliness and depressive symptoms, including suicidal ideation [29]. Another study acknowledged the positive correlation between social isolation and mental health (symptoms of anxiety and depression) in patients with breast cancer [30]. Supporting existing evidence, our study observed a significant direct effect, where increased social isolation was responsible for worsening the mental health of patients with cancer. Adding to the body of knowledge, we also found a significant indirect effect of social isolation on mental health, that is, a significant mediation effect of negative self-perception. It is worth noting that most of these findings are consistent with previous research, but discrepancies may arise because of differences in the type of cancer or the stage of the illness.

Another novelty of our study is the quadratic (curvilinear) effect of social isolation on the mental health of patients with cancer. This finding, as indicated by a statistically significant coefficient of −0.136, suggests that the relationship between the 2 variables is not necessarily a simple linear relationship; rather, it follows a more complex pattern. The negative coefficient (−0.136) for the quadratic term indicates that the relationship between social isolation and mental health is stronger at very high or very low levels of social isolation and weaker at moderate levels. This finding suggests that there may be a threshold level of social isolation beyond which mental health outcomes deteriorate more rapidly. Therefore, minimizing social isolation might not be optimal for reducing mental distress among patients with cancer. Instead, attempts should be made to provide moderate isolation to the patients where they stay, with adequate time for self-reflection and access to social activities.

The linear term (−0.321) represents the overall trend in the relationship between social isolation and mental health, with a negative coefficient indicating that mental health decreases as social isolation increases. This finding is consistent with the idea that social isolation is generally associated with negative mental health outcomes. However, the magnitude of this effect may vary, depending on the level of social isolation. Overall, these findings suggest that social isolation is an important factor to consider in the mental health of patients with cancer and that interventions to reduce social isolation may be an effective way to improve mental health outcomes in this population. Further research is needed to understand the nature of the relationship between social isolation and mental health in patients with cancer, as well as the potential moderating or mediating factors that may influence this relationship. It is also important to consider the implications of these findings for clinical practice; for example, health care providers may need to pay particular attention to the social isolation levels of patients with cancer and address any potential issues that may arise. This may involve providing support and resources to help patients maintain social connections or referring patients to social workers or other professionals who can provide additional support.

The finding of a significant effect of negative self-perception on mental health, as indicated by a coefficient of −0.22 and a statistically significant *P* value, suggests that self-perception is a crucial determinant of mental health in patients with cancer. The negative coefficient for the effect of negative self-perception on mental health indicates that lower levels of negative self-perception are associated with better mental health outcomes. This finding suggests that self-perception may have a protective effect on mental health in patients with cancer, potentially by providing individuals with a more positive view of themselves and their abilities. Consistent with our findings, a 2021 study reported a significant association between self-perception and mental health in patients with cancer [18]. Another study in 2017 found a relationship between negative self-perception and deteriorated physical and mental health [31]. This longitudinal study observed 100 patients with cancer and followed the relationship between self-perception and mental health for a year [31].

In our study, physician-patient communication was found to be positively associated with mental health, suggesting that patients with cancer who reported higher levels of satisfaction with their communication with their physicians had higher levels of mental health. This is consistent with research showing that effective physician-patient communication is important in promoting mental health [32]. However, our study is the first to explore this relationship explicitly for patients with cancer. Effective communication can expedite patient recovery [33], not necessarily as a direct effect but possibly via indirect routes such as establishing physician-patient trust, understanding, and agreement. Ultimately, these proximal outcomes lead to overall well-being through improved access to care, better patient knowledge, shared decision-making, management of emotions, and patient empowerment. The positive impact of physician-patient communication on patients’ self-perception, as observed in our study, has also been noted by others in a different context. A 2022 study reported the positive impact of effective physician-patient communication on patients’ perception of safety and security, thereby augmenting their self-perception [34]. Another study in 2020 dealing with 250 patients with hypertension reported a significant impact of effective physician-patient communication on patients’ perception of self-care ability and satisfaction as well as pharmaceutical adherence in patients with hypertension [35]. These findings support the notion that improving physician-patient communication may enhance self-perception in patients with cancer.

Our study observed that the magnitude of the indirect impact of social isolation on mental health, when mediated by negative self-perception, was significantly greater in patients with cancer who were treated before the COVID-19 pandemic than in those who received treatment during the pandemic. This implies that negative self-perception played a much stronger role in determining the mental health of patients with cancer before the pandemic. It is also important to note that the relationships between these variables may vary based on patient characteristics such as age, literacy, and income; for example, older patients with cancer may be more vulnerable to social isolation because of a higher prevalence of physical limitations and a smaller social network. By contrast, younger patients with cancer may be more vulnerable to social isolation because of a lack of experience with illness and a greater reliance on social support.

Similarly, patients with cancer with lower literacy levels may have difficulty understanding medical information and experience poorer communication with their health care providers. This may affect their mental health outcomes. Finally, patients with cancer with lower incomes may have limited access to health care and may experience financial stress, which can affect their mental health.

### Implications and Limitations

Our findings have important implications for the overall treatment of patients with cancer. They suggest that interventions addressing social isolation, improving self-perception, and enhancing physician-patient communication may improve the mental health of patients with cancer. These interventions could include support groups, cognitive behavioral therapy, and training for health care providers in effective communication skills. Future research should examine the effectiveness of these interventions in different subgroups of patients with cancer, such as those with different ages, literacy levels, and income levels.

It is important to acknowledge the limitations of this study. One potential limitation is that the data were collected through self-report measures subject to biases such as social desirability and self-presentation. In addition, the results may not be generalizable to the larger population of patients with cancer. It would be valuable to replicate this study with a larger sample to further examine the relationships among the variables. Furthermore, the study was conducted during a particularly fluid period when social isolation was the norm because of the COVID-19 pandemic. It is important to consider whether the curvilinear relationship between social isolation and mental health observed in our study will hold true once the pandemic has fully resolved. Although we acknowledge that the long-term relevance of our findings in nonpandemic contexts is uncertain, the curvilinear relationship between social isolation and mental health observed in our study is an important finding that may have implications beyond the current pandemic. Future research should explore the impact of social isolation on mental health in patients with cancer in nonpandemic contexts, which may help to elucidate further the complex relationship between social isolation and mental health. Despite the uncertainties surrounding the long-term relevance of our findings, we believe that our study provides valuable insights into the impact of social isolation on the mental health of patients with cancer, particularly during times of heightened social isolation, such as during the COVID-19 pandemic.

## Abbreviations

AVE: average variance extracted
BCA: bias-corrected and accelerated
GAD-2: 2-item Generalized Anxiety Disorder scale
HINTS: Health Information National Trends Survey
HTMT: heterotrait-monotrait
MGA: multigroup analysis
NCI: National Cancer Institute
PHQ-2: 2-item Patient Health Questionnaire
PHQ-4: 4-item Patient Health Questionnaire
PLS-SEM: partial least squares structural equation modeling
PPC: physician-patient communication
PROMIS: Patient-Reported Outcomes Measurement Information System
QE: quadratic effect
SEER: Surveillance, Epidemiology, and End Results
SEM: structural equation modeling
SRMR: standardized root mean square residual
VIF: variance inflation factor

## Data Availability

The study does not contain identifiable patient data. Consent from the individuals involved in this study was not required. The author cannot grant requests for any underlying data because the data were acquired from the Health Information National Trends Survey (HINTS) under the Microdata Dissemination Policy. Individuals and organizations wishing to access the data can make a request directly to the HINTS service.
